# Exploring the multigenerational effects of *Lippia adoensis* (Lamiales: Verbenaceae) essential oil and Dichlorvos on *Callosobruchus maculatus* (Coleoptera: Bruchidae) life-history traits and gene expression

**DOI:** 10.1093/jisesa/ieag107

**Published:** 2026-09-29

**Authors:** Mazarin Akami, Wei Jiang, Bingbing Wei, Ousman Tamgue, Cabrel Bafong Ngueya, Diane Pocssie Samenoug, Francioli Koro Koro, Rosalie Annie Ngono Ngane, Nicolas Njintang Yanou, Elias Nchiwan Nukenine, Baolei Jia, Yanlai Yao, Chang-Ying Niu

**Affiliations:** Department of Biochemistry, Faculty of Science, University of Douala, P.O. Box 24, 157, Douala, Cameroon; Xianghu Laboratory, Hangzhou, 311231, China; Xianghu Laboratory, Hangzhou, 311231, China; College of Plant Science & Technology, Huazhong Agricultural University, No. 1 Shizishan Street, Wuhan 430070, China; Department of Biochemistry, Faculty of Science, University of Douala, P.O. Box 24, 157, Douala, Cameroon; Department of Biochemistry, Faculty of Science, University of Douala, P.O. Box 24, 157, Douala, Cameroon; Department of Biochemistry, Faculty of Science, University of Douala, P.O. Box 24, 157, Douala, Cameroon; Department of Biochemistry, Faculty of Science, University of Douala, P.O. Box 24, 157, Douala, Cameroon; Department of Biochemistry, Faculty of Science, University of Douala, P.O. Box 24, 157, Douala, Cameroon; Department of Biological Sciences, Faculty of Science, University of Ngaoundere, Ngaoundere, Cameroon; Department of Food Science and Nutrition, National School of Agro-Industrial Sciences, University of Ngaoundere, Ngaoundere, Cameroon; Department of Biological Sciences, Faculty of Science, University of Ngaoundere, Ngaoundere, Cameroon; Xianghu Laboratory, Hangzhou, 311231, China; Xianghu Laboratory, Hangzhou, 311231, China; College of Plant Science & Technology, Huazhong Agricultural University, No. 1 Shizishan Street, Wuhan 430070, China

**Keywords:** *Callosobruchus maculatus*, Dichlorvos, *essential oil*, detoxification enzyme, transcriptional response, *Callosobruchus maculatus*, Dichlorvos, *huiles essentielle*, enzymes de détoxication, réponse transcriptionnelle

## Abstract

The cowpea weevil, *Callosobruchus maculatus* (Fabricius) (Coleoptera: Bruchidae), is a major stored-product pest. Resistance to synthetic insecticides such as dichlorvos (DDVP) has prompted interest in alternatives with distinct modes of action. This study compared multigenerational transcriptional responses of *C. maculatus* to DDVP and the botanical insecticide *Lippia adoensis* (Hochst. ex Walp.) (Lamiales: Verbenaceae) essential oil (EO). DDVP exposure was associated with progressive fitness recovery across generations, together with upregulated expression of detoxification-related genes (e.g. *Cyp6g1-2*, *Cyp6g1-3*, *Gst*, *Est2*, *Pod*) and cholesterol metabolism-related genes (e.g. *Dhcr24-1*, *Dhcr24-3*). In contrast, *L. adoensis* EO was associated with consistently higher mortality and did not elicit comparable transcriptional changes in these gene sets. Clustering patterns placed EO-treated populations closer to controls, with less evidence of the DDVP-associated expression pattern. An antagonistic index comparing treatment-related fold changes indicated an opposing transcriptional relationship between EO and DDVP. Overall, the findings are consistent with *L. adoensis* EO acting without inducing the DDVP-associated detoxification expression pattern, suggesting a distinct transcriptional profile that may inform botanical-based approaches to pest management.

## Graphical Abstract

**Graphical Abstract:**
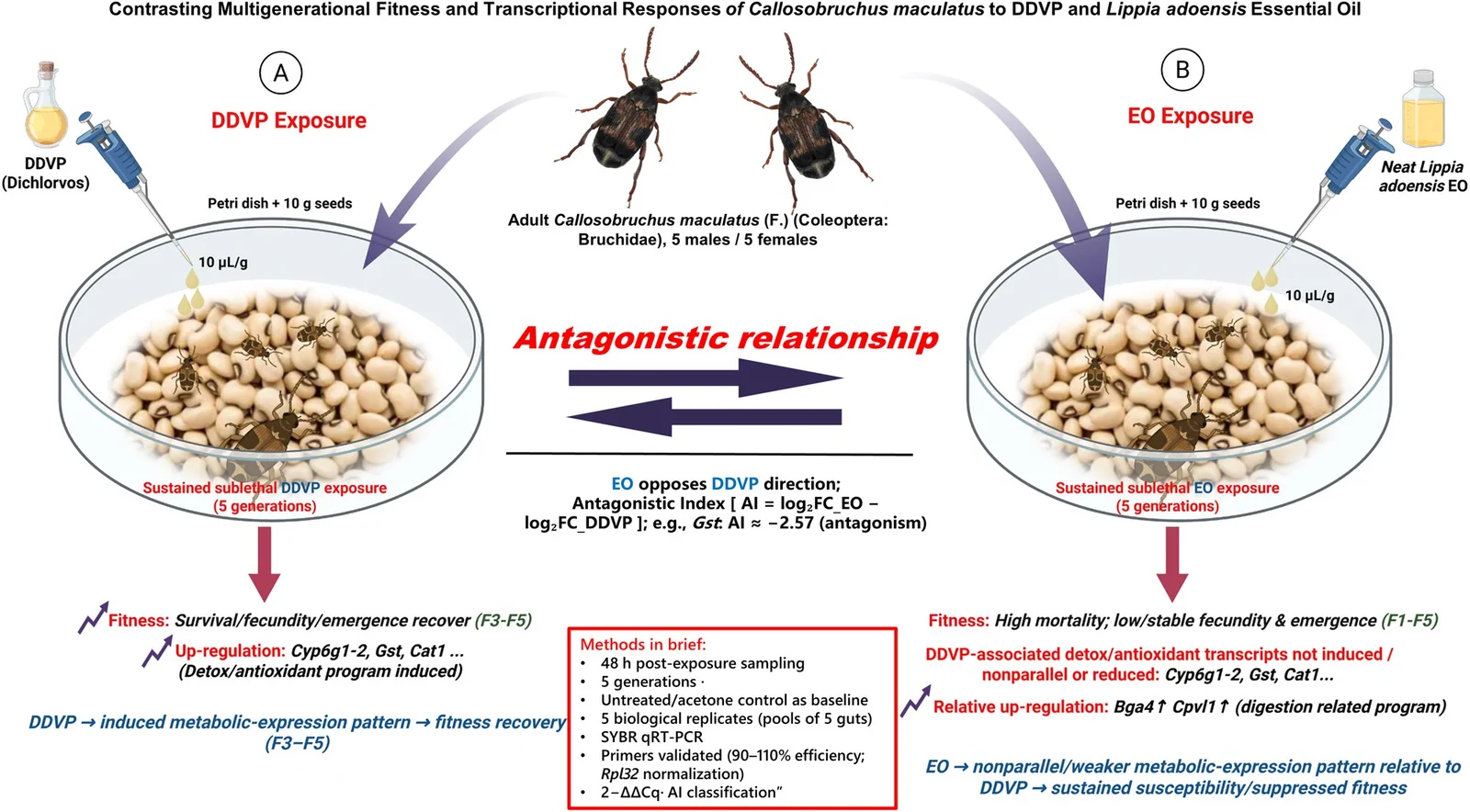

## Introduction

The evolution of resistance to synthetic pesticides represents a major constraint in modern pest management, undermining global food security and increasing production costs. Metabolic resistance is particularly common and is often associated with overexpression of enzyme families such as cytochrome P450 monooxygenases (P450s), glutathione S-transferases (GSTs), and esterases, which convert lipophilic insecticides into excretable compounds (Whalon et al. 2008, Feyereisen 2012, Montella et al. 2012). Detoxification processes are often organized in phases: phase I, oxidation by P450s, phase II conjugation by GSTs, and concurrent mobilization of antioxidant defenses such as catalases and peroxidases to mitigate oxidative stress (Song et al. 2022, Garbaly et al. 2025, Wu et al. 2025). The transcriptional regulation of these enzymes can involve pathways such as CncC/Keap1 (Amezian et al. 2021, Li et al. 2021).

*Callosobruchus maculatus* F. (Coleoptera: Bruchidae), the cowpea weevil, is a damaging stored-product pest capable of causing substantial post-harvest losses in cowpea (Beck and Blumer 2011, Akami et al. 2017). Its short life cycle and internal seed development make control difficult. Although transcriptomic studies have begun to characterize gene activity in this species (Sayadi et al. 2016, Arnqvist et al. 2024), the molecular basis of insecticide response in *C. maculatus* remains limited.

Dichlorvos (DDVP), an organophosphate that inhibits acetylcholinesterase, has been widely used against stored-product pests, yet reduced susceptibility is increasingly reported (Omoniyi et al. 2013, Gbaye et al. 2016). This has increased interest in botanical alternatives with distinct modes of action. Plant essential oils (EOs), especially those rich in monoterpenoids, can affect insect behavior and physiology and may modulate detoxification-related gene expression at sublethal doses (Tak and Isman 2017, Gupta et al. 2023).

*Lippia adoensis* (Hochst. ex Walp.) (Lamiales: Verbenaceae), native to East Africa and traditionally used in stored-grain protection, produces an EO containing compounds such as carvacrol/thymol-related constituents and *p*-cymene, with reported insecticidal and enzyme-modulating properties (Adelani et al. 2016, Majolo et al. 2017). Prior work has documented activity against *C. maculatus* (Akami et al. 2017). These features make *L. adoensis* EO an ideal candidate for testing whether a botanical exposure is associated with a different transcriptional profile than DDVP.

To compare molecular responses under chronic exposure, we focused on 14 target genes grouped into 4 functional categories: (i) detoxification/metabolism-related genes (*Cyp6g1-2*, *Cyp6g1-3*, *Gst*, *Est2*, *Pod*); (ii) antioxidant defense (*Cat1*, *Cat2*, *Hmt*); (iii) digestion and metabolism (alpha-amylase [*Amy1*], beta-galactosidase [*Bga4*], carnosine/anserinase [*Cpvl1*], cobalamin synthase [*Cbl2*]); and (iv) lipid and cholesterol metabolism (*Dhcr24-1*, *Dhcr24-3*). These were selected because they are known or hypothesized targets to respond to insecticide or plant-defensive-compound exposure, and their differential regulation could be associated with the observed fitness differences.

Our study aimed to compare multigenerational fitness and transcriptional responses of *C. maculatus* exposed to DDVP versus *L. adoensis* EO and to assess whether treatment-specific gene expression patterns are consistent with an antagonistic transcriptional relationship. Specifically, we addressed whether (i) fitness trajectories diverge across generations under sustained sublethal DDVP versus EO exposure; (ii) detoxification, antioxidant, digestive, and lipid/metabolism genes show distinct expression dynamics under the 2 treatments; and (iii) EO exposure is associated with reduced induction of DDVP-associated metabolic transcripts. We hypothesized that DDVP would be associated with upregulation of metabolic/resistance-associated genes, whereas *L. adoensis* EO would show weaker or nonparallel induction of these pathways.

## Materials and Methods

### Procurement of Plant Materials and EO

Mature, uninfested cowpea seeds (*Vigna unguiculata* L. Walp) (Fabales: Fabaceae) of a local susceptible variety were sourced from a local market. Seeds were visually inspected, cleaned, and stored at 4 °C until use. Fresh aerial parts (leaves and stems) of *L. adoensis* (Hochst. ex Walp.) were collected from their natural habitat. The plant material was taxonomically identified and a voucher specimen (voucher number: XYZ123) was deposited at the National Herbarium. The EO was extracted from 500 g of shade-dried, coarsely ground plant material via hydrodistillation for 3 h using a Clevenger-type apparatus. The obtained pale-yellow EO was dried over anhydrous sodium sulfate, filtered, and stored in sealed amber glass vials at 4 °C until used for bioassays. The chemical composition of *L. adoensis* EO was characterized by gas chromatography-mass spectrometry (GC-MS) using an INNOWax column. The oil was diluted in an appropriate solvent and analyzed in full-scan mode. Compounds were identified by comparison of retention indices and mass spectra with reference libraries (NIST). Relative percentages were calculated from peak areas. A summary of the major constituents is presented in Table 1. This analysis, performed as previously reported, confirmed the major constituents of the EO to be thymol, thymol acetate, p-cymene, and triacetin, which aligns with its documented insecticidal activity (Akami et al. 2016).

**Table 1.** Major constituents of *Lippia adoensis* essential oil (young leaves) (≥2% relative abundance)

No.	Compound	Class	Retention time (min)	Relative content (%)
**1**	Thymol	Oxygenated monoterpene	51.98	22.02
**2**	Thymol acetate	Oxygenated monoterpene	41.03	15.21
**3**	*para*-Cymene	Monoterpene hydrocarbon	17.33	13.85
**4**	Triacetin	Hydrocarbon derivative	48.59	9.13
**5**	γ-Terpinene	Monoterpene hydrocarbon	16.13	6.42
**6**	Caryophyllene	Sesquiterpene hydrocarbon	31.29	3.82
**7**	3-Aminopyrazole	Hexane and octane derivative	31.05	3.30
**8**	Carvacrol	Oxygenated monoterpene	39.84	3.26
**9**	Limonene	Monoterpene hydrocarbon	13.47	3.36
**10**	β-Farnesene	Sesquiterpene hydrocarbon	34.67	2.88
**—Other constituents (33 compounds)—**				18.85
**Total identified**				93.54

Full constituent list including retention indices, match factors, and identification methods is provided in Table S4.

### Insect Rearing and Experimental Design

A wild population of *C. maculatus* was established from infested cowpea seeds obtained from traditional storage facilities. Insects were reared on untreated, clean cowpea (*V. unguiculata*) seeds in 1-l transparent glass jars under controlled conditions (27 ± 2 °C, 65% ± 5% relative humidity, 14:10 h light:dark photoperiod). Jar tops were covered with muslin cloth for ventilation. Under these conditions, the life cycle from egg to adult was approximately 28 to 30 days. The colony was maintained for several generations to obtain a synchronized, susceptible population. For experiments, newly emerged adults (1 to 2 days old) were allowed to mate prior to use.

### Treatment Preparation and Exposure

Cowpea seeds were disinfested by freezing at −20 °C for 7 days prior to use. Three treatment groups were established: (i) “DDVP group,” seeds treated with a commercial DDVP formulation at 10 µl/g of seed, a sub-lethal dose established for *C. maculatus* (Akami et al. 2017); (ii) “EO group,” seeds treated with neat *L. adoensis* EO at the same volumetric concentration (10 µl/g) to enable direct comparison; and (iii) “Control group,” seeds treated with an equivalent volume of acetone carrier solvent. Each treatment was applied evenly onto the seed surface using a micropipette, and the solvent was allowed to evaporate for 15 min under a fume hood before introducing insects.

For each treatment and generation (F1 to F5), 5 independent replicates were established. Each replicate consisted of 10 g of treated cowpea seeds placed in a 90 mm diameter Petri dish, into which 10 adult *C. maculatus* (5 males, 5 females; 1 to 2 days old, mated) were introduced (*n* = 50 per treatment per generation; 5 biological replicates × 10 individuals per replicate). Dishes were maintained in a controlled environment growth chamber (27 ± 1 °C, 65% ± 5% RH, 14:10 h L:D photoperiod) for a total exposure period of 6 days (Digilio et al. 2008, Chakira et al. 2017).

### Assessment of Fitness Parameters

- **Cumulative adult mortality:** The number of dead adults in each Petri dish was recorded at 1-, 2-, 4-, and 6-days post-exposure (dpe). Mortality at 6 dpe was used as the final measure for that generation and expressed as a percentage of the initial 10 adults per replicate. Dead insects were removed upon counting. Survival (%) was calculated as 100 − mortality (%).
- **Fecundity:** At the end of the 6 dpe period, all insects (live and dead) were removed. The total number of eggs laid on the seeds in each Petri dish was counted under a stereomicroscope, and dishes were returned to the growth chamber. Fecundity was expressed as the mean number of eggs laid per female during the exposure period per replicate.
- **Pupal eclosion:** At 48 h post-oviposition, eggs were counted under a stereomicroscope with backlight illumination to enhance contrast between eggs and the cowpea seed coat. The number of adults that successfully emerged from the seed-coated eggs was recorded daily per replicate. The emergence success rate (fertility) was calculated as: (number of emerged adults/total number of eggs) × 100.

#### Establishment of Subsequent Generations and Resistance Monitoring

To study the multigenerational response, the protocol was iterative. For each treatment group, the seeds containing the offspring (larvae/pupae) from the surviving insects of 1 generation (e.g. F1) were pooled and allowed to develop. Upon emergence, the new adults (constituting the F2 generation for that treatment lineage) were collected, and the entire bioassay procedure, from seed treatment to fitness assessment, was repeated identically. This process was continued sequentially until the fifth generation (F5). Mortality was tracked across generations to characterize changes in treatment response over time.

#### Gene Selection and Functional Classification

Fourteen target genes were selected to represent 4 major meta­bolic functional categories. Five genes were classified as detoxification genes: 2 cytochrome P450 monooxygenases (*Cyp6g1-2*, *Cyp6g1-3*), 1 *Gst*, 1 esterase (*Est2*), and 1 peroxiredoxin/peroxidase (*Pod*). Three genes were classified as antioxidant defense genes: 2 catalases (*Cat1*, *Cat2*) and 1 hydroxymethyltransferase (*Hmt*). Four genes were classified as digestion/metabolism genes: *Amy1*, *Bga4*, *Cpvl1*, and *Cbl2*. The remaining 2 genes, *Dhcr24-1* and *Dhcr24-3*, are paralogous 24-dehydrocholesterol reductases and were grouped as lipid/cholesterol metabolism genes. This functional classification guided both the Quantitative Real-Time PCR (qRT-PCR) assay design and the downstream visualization of expression data.

#### Gene Expression Profile by qRT-PCR in Response to DDVP and EO Treatments

##### Insect dissection and RNA extraction

To analyze the molecular response, gene expression was profiled across 5 generations (F1 to F5) for each treatment (DDVP, EO, and Control). For each generation and treatment, 5 biological replicates were established. For each replicate, 5 newly emerged adults (1 to 2 days old) were randomly selected, surface-sterilized, and dissected aseptically under a stereomicroscope (Pitsch et al. 2025). The entire gut (including the foregut, midgut, and hindgut) was isolated, rinsed in ice-cold Phosphate-Buffered Saline (PBS) (pH 7.4), and immediately transferred to a sterile 1.5 ml microcentrifuge tube containing 750 µl of TRIzol reagent (Invitrogen, United States). Total RNA was extracted following the manufacturer’s standard protocol. The extracted RNA was treated with DNase I (Thermo Fisher Scientific, United States) to eliminate potential genomic DNA contamination (Hussain et al. 2026). RNA concentration and purity were assessed using a NanoDrop 2000 spectrophotometer (Thermo Fisher Scientific, United States), with acceptable 260/280 and 260/230 ratios >1.8. RNA integrity was verified by 1.5% agarose gel electrophoresis, visualizing clear 18S and 28S ribosomal RNA bands.

##### cDNA synthesis

For first-strand complementary DNA (cDNA) synthesis, 1 µg of total RNA from each sample was reverse-transcribed using the PrimeScript RT reagent Kit with gDNA Eraser (Takara Bio, Japan) according to the manufacturer’s instructions. The reaction mix included gDNA Eraser, random hexamer primers, and PrimeScript RT Enzyme Mix. The thermocycling conditions were: 37 °C for 15 min (gDNA removal), 85 °C for 5 s (enzyme inactivation), and 4 °C hold. The resulting cDNA was diluted 1:5 with nuclease-free water and stored at −20 °C until use in qPCR. The RNA extraction and cDNA synthesis workflow is shown in Supplementary Fig. S1.

##### Primer design and qRT-PCR

Gene-specific primers were designed from *C. maculatus* transcriptome sequences (Benton et al. 2016, Sayadi et al. 2016, Arnqvist et al. 2024). Nucleotide sequences of the 14 target genes were retrieved from NCBI GenBank. Specific and intron-spanning primer pairs were designed using the Primer-BLAST tool (https://www.ncbi.nlm.nih.gov/tools/primer-blast/) (Yang et al. 2023) with the following parameters: amplicon size 80 to 150 bp, primer melting temperature of 55 to 60 °C, and a GC content of 40% to 60%. Primer specificity was confirmed in silico by BLASTn against the *C. maculatus* transcriptome and experimentally verified by melt-curve analysis and gel electrophoresis. PCR efficiency for all primer pairs was validated to fall within 90% to 110%. The primer sequences, amplicon sizes, and calculated PCR efficiencies are detailed in Supplementary Table S1. Cloning and sequencing procedures are described in online supplementary data, Method S1.

The expression stability of 2 commonly used reference genes, ribosomal protein L32 (*Rpl32*) and Elongation factor 1-alpha (*Ef1α*), was evaluated across all samples using the geNorm algorithm (Vandesompele et al. 2002). Based on stability analysis (M value < 0.5), *Rpl32* was selected as the optimal endogenous control for normalization of target gene expression data (*Ef1α* was discarded). For each treatment and generation, 5 biological replicates were analyzed, each consisting of pooled RNA from 5 individuals (*n* = 5 biological replicates, each a pool of 5 insect guts; 25 insects total). Quantitative RT-PCR was performed using SYBR Green chemistry on a real-time PCR system.

##### qRT-PCR procedures

qRT-PCR was performed on an ABI 7300 Real-Time PCR System (Applied Biosystems, United States) using TB Green Premix Ex Taq II (Tli RNaseH Plus) (Takara Bio, Japan). Each 20 µl reaction contained TB Green Premix, diluted cDNA template, gene-specific primers, and nuclease-free water. Thermal cycling conditions were: 95 °C for 30 s, followed by 40 cycles of 95 °C for 5 s, and 55 °C for 30 s. A melt-curve analysis was performed to confirm amplicon specificity. No-template and no-reverse-transcriptase controls were included to verify reagent integrity and absence of genomic DNA contamination. All samples (5 biological replicates per treatment per generation, each derived from a pool of 5 insect guts; 25 insects total) were assayed with technical duplicates on the same plate to minimize inter-plate variability. Amplification efficiencies (90% to 110%) were determined from a standard curve generated from a pooled cDNA serial dilution. The complete reaction compositions and cycling parameters are provided in Supplementary Table S2.

##### Quantification of gene expression

Gene expression was assessed at 48 h post-exposure, a time point selected based on preliminary time-course experiments showing peak transcriptional response for detoxification genes (data not shown). The quantification cycle (Cq) values were determined using the instrument’s software with the default threshold setting. Technical duplicate Cq values were averaged before further calculation. The relative expression level of each target gene was calculated using the comparative 2^(−ΔΔCq) method (Livak and Schmittgen 2001). First, the ΔCq for each sample was calculated as Cq (target gene) − Cq (reference gene, *Rpl32*). Then, ΔΔCq was calculated as ΔCq (treatment sample) − ΔCq (mean of Control group samples for the corresponding generation). The resulting ΔΔCq values for all biological replicates (*n* = 5 pools per treatment per generation, each pool derived from 5 insect guts; 25 insects total) were exported for statistical analysis, as described in the following section.

##### Antagonistic index calculation and interpretation

The Antagonistic Index (AI) was calculated to compare the transcriptional response between EO and DDVP treatments for each target gene. Fold changes (FCs) were first computed using the 2^(−ΔΔCq) method, ensuring all FC values were ≥0. The AI was then defined as:


$$\text{AI}={\;\;\text{log}\;}_{2}\left({\text{FC}}_{EO}\right)-{\;\;\text{log}\;}_{2}\left({\text{FC}}_{\mathrm{DDVP}}\right)$$

where FC_EO_ and FC_*DDVP*_ represent the FC in gene expression relative to the Control under EO and DDVP treatments, respectively, calculated using the 2^(−ΔΔCq) method. Negative AI values indicate that EO and DDVP push gene expression in opposite directions (antagonism), while positive values indicate they push in the same direction (co-induction). The magnitude of AI determined the strength classification: |AI| > 1.5 = Strong, 0.5 to 1.5 = Moderate, and <0.5 = Weak. The step-by-step calculation of the AI for target genes is provided in Supplementary Table S3.

#### Data Analyses and Visualization

All data for fitness parameters (cumulative survival, fecundity, and fertility) and gene expression (ΔΔCq values) were recorded in Microsoft Excel 365. Normality (Shapiro–Wilk) and homogeneity of variances (Levene’s test) were verified; percentage data were arcsine square-root transformed where necessary. To evaluate overall treatment effects across all generations, 1-way ANOVA with Treatment (Control, EO, DDVP) as the fixed factor was performed, followed by Tukey’s HSD post-hoc test. To assess temporal dynamics across generations, a 2-way repeated measures ANOVA (RM-ANOVA) was conducted using GraphPad Prism (version 9.5.1, GraphPad Software, San Diego, California, United States) with a significance threshold of α = 0.05. Treatment and Generation were included as fixed factors, and Replicate served as the subject variable. Where sphericity assumptions were violated, the Geisser–Greenhouse correction was applied. Following a significant interaction term, Tukey’s HSD test was used for post-hoc pairwise comparisons. Results are reported as *F*-values with degrees of freedom and exact *P*-values.

##### Fitness data

Means are presented with SEM (*n* = 50 per treatment per generation; 5 biological replicates × 10 individuals per replicate).

##### Gene expression data

For each treatment and generation, 5 biological replicates were analyzed, each consisting of pooled RNA from 5 individuals (*n* = 5 pools per treatment per generation, each pool derived from 5 insect guts; 25 insects total). All biological replicates and technical duplicates were run on the same plate to minimize inter-plate variability. The quantitative analysis of antagonistic gene regulation between EO and DDVP treatments across 5 generations is presented in Table 2.

Graphical representations were created using GraphPad v9.5.1 software, with error bars representing SEM. The heatmap illustrating differential gene expression profiles (FCs relative to the control) was constructed using TBtools-II v2.0.0 software (Chen et al. 2023), with hierarchical clustering of both genes and treatments/generations based on Euclidean distance and complete linkage. Row annotation bars indicated functional gene categories; column annotation bars indicated treatment groups. Values shown are ΔΔCq (equivalent to log₂ FC relative to Control). Red indicates upregulation; blue indicates downregulation. The graphical abstract was designed using Microsoft PowerPoint 365, incorporating icons from BioRender.com and The Noun Project under academic license. All analyses adhered to MIQE guidelines (Bustin 2024).

## Results

### Chemical Composition of *L. adoensis* EO

GC-MS analysis of *L. adoensis* young leaves EO identified 43 compounds, representing 93.54% of the total oil composition (Table 1). The oil is dominated by thymol (22.02%), thymol acetate (15.21%), para-cymene (13.85%), and triacetin (9.13%). Other notable constituents included γ-terpinene (6.42%), caryophyllene (3.51%), limonene (3.21%), carvacrol (3.26%), and β-farnesene (2.64%). The complete chromatographic profile, including retention times, group assignments, and all 43 compounds is presented in Supplementary Table S4.

### Multigenerational Impact on Survival, Fecundity, and Fertility (the Phenotype)

The fitness of *C. maculatus* populations was assessed over 5 consecutive generations (F1 to F5) under continuous sublethal pressure from DDVP and *L. adoensis* EO. Across the 3 key fitness parameters, the synthetic insecticide DDVP and the botanical *L. adoensis* EO exhibited fundamentally different multigenerational profiles. One-way ANOVA revealed a significant treatment effect on overall survival (*F*_(2,72)_ = 33.971, *P *< 0.0001), fecundity (*F*_(2,72)_ = 212.535, *P *< 0.0001), and pupal eclosion (*F_(2,72)_* = 98.473, *P *< 0.0001). For survival, both EO and DDVP differed significantly from the Control (both *P* < 0.0001), but not from each other (*P *= 0.187). For fecundity and pupal eclosion, all pairwise comparisons between treatments were significant (all ns, *P *< 0.0001).

#### Adult Survival

One-way ANOVA revealed a significant overall treatment effect (*F*_(2,72)_ = 33.97, *P *< 0.0001). Both EO and DDVP differed significantly from the Control (***P < 0.0001), but not from each other (ns, *P *= 0.187), reflecting their distinct temporal dynamics rather than equivalent net effects (Fig. 1).

**Fig. 1.**
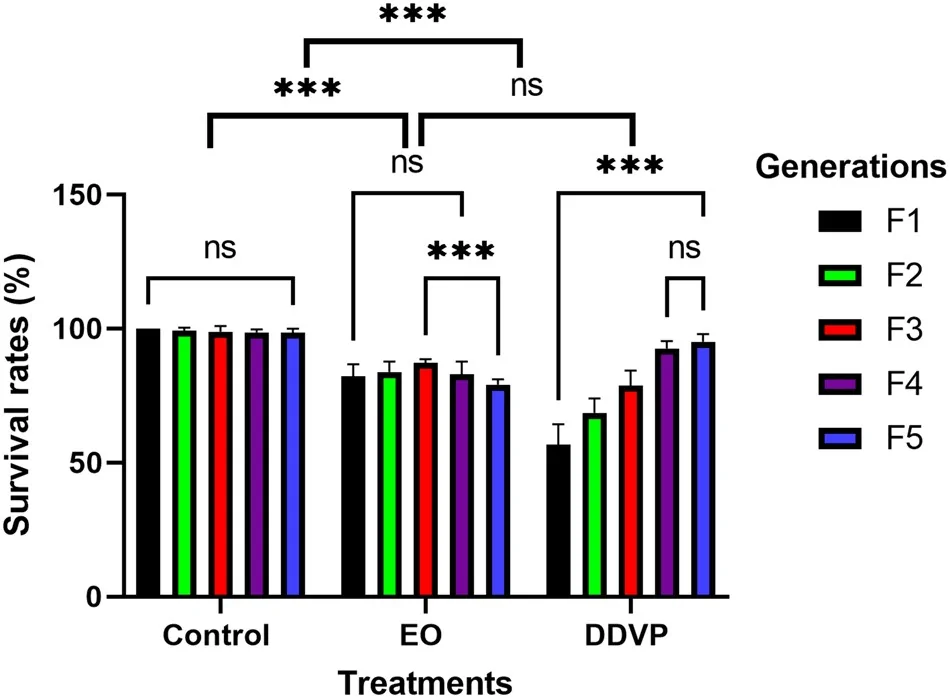
Survival of *Callosobruchus maculatus* across 5 generations under dichlorvos (DDVP), *Lippia adoensis* essential oil (EO), and control treatments. Data are mean ± SEM (*n *= 50 per treatment per generation). Brackets indicate significant differences between treatments (1-way ANOVA, *F*_(2,72)_ = 33.971, *P *< 0.001). Brackets with asterisks (***) denote significant generational differences within treatments (2-way repeated-measures ANOVA, Tukey HSD, *P *< 0.001); ns = non-significant. Control and EO groups remained stable across generations; DDVP showed a significant upward trend from F1 to F5.

To assess temporal dynamics, 2-way RM-ANOVA revealed a highly significant Treatment × Generation interaction (*F*_(8,40)_ = 29.97, *P *< 0.0001, Geisser–Greenhouse correction), along with significant main effects for Treatment (*F*_(4,20)_ = 42.05, *P *< 0.0001) and Generation (*F*_(1.48,29.52)_ = 179.2, *P *< 0.0001). Tukey’s HSD post-hoc test indicated stable survival across generations in the Control group (all ns, *P *> 0.05). The EO group remained stable except for a significant decline between F3 and F5 (***P = 0.0009). In contrast, the DDVP group showed a strong initial fitness cost (F1: ∼56%) followed by progressive recovery to ∼95% by F5, with significant increases between F1 vs. F4/F5 and F2 vs. F4/F5 (all ***P < 0.001), suggesting tolerance development over successive generations. The F4 to F5 comparison was non-significant (ns, *P *> 0.05), indicating a plateau.

#### Fecundity

A significant Treatment × Generation interaction was detected (RM-ANOVA: *F_(1.434,5.736)_* =10.32, *P *= 0.0155), with significant main effects for Generation (*F_(1.000,4.000)_* =71.27, *P* < 0.0001) and Treatment (*F_(1.530,6.120)_* =6.685, *P *= 0.0332). Stable fecundity across generations was recorded in both Control and EO groups (Tukey’s test, all *P *> 0.05). Conversely, the DDVP group exhibited a significant progressive increase in fecundity. F1 was significantly lower than F3 (*P *= 0.0002), F4 (*P *= 0.0278), and F5 (*P *= 0.0029), while F2 was significantly lower than F5 (*P *= 0.0337). This upward trend suggests recovery or development of tolerance in reproductive output under continued DDVP exposure (Fig. 2A).

**Fig. 2.**
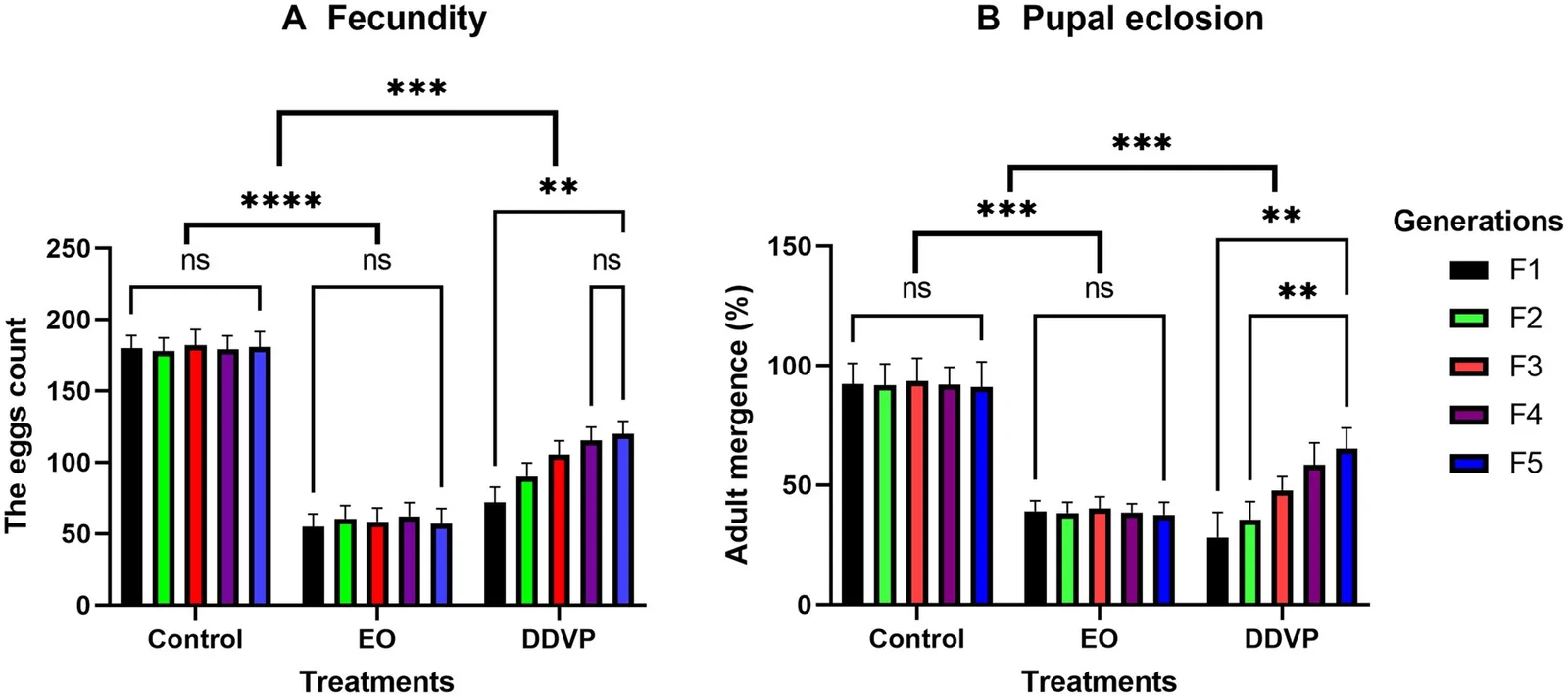
Fecundity and pupal eclosion of *Callosobruchus maculatus* across 5 generations under control, EO, and DDVP treatments. A) Mean number of eggs laid per female. B) Mean adult emergence (%). Data are mean ± SEM (*n *= 50 per treatment per generation). Asterisks (**) indicate significant differences between treatments (1-way ANOVA, *P *< 0.05). Asterisks (***;****) denote significant generational differences within treatments (2-way repeated-measures ANOVA, Tukey HSD, ***P < 0.001; **** *P* < 0.0001); ns = non-significant. Control and EO groups remained stable; DDVP showed significant recovery of both traits.

#### Fertility (Pupal Eclosion)

Similarly, a significant Treatment × Generation interaction was observed (RM-ANOVA: *F*_(1.473,5.891)_ = 8.230, *P *= 0.0235, Geisser–Greenhouse correction), with significant main effects for Generation (*F*_(1.572,6.289)_ = 16.93, *P *< 0.0001) and Treatment (*F*_(1.898,7.593)_ = 6.320, *P *= 0.0251). Tukey’s test indicated stable eclosion across generations in both the Control (∼90%) and EO (∼35% to 40%) groups (all *P *> 0.05). In contrast, the DDVP group showed significant recovery over time. F1 and F2 exhibited severely suppressed emergence (∼25% and ∼33%, respectively), with significant improvements by F4 (∼57%) and F5 (∼63%) (F1 vs. F4, *P *= 0.0076; F1 vs. F5, *P *= 0.0077; F2 vs. F5, *P *= 0.0022). By F5, eclosion reached ∼63%, suggesting tolerance development over successive generations (Fig. 2B).

#### Summary of Fitness Costs

Multigenerational exposure revealed contrasting adaptive responses. *L. adoensis* EO exerted a sustained suppressive effect on both fecundity and fertility across all generations. In contrast, DDVP initially imposed severe reproductive costs, but the population recovered significantly from F3 generation onward, indicating tolerance development. This contrast underscores the botanical’s advantage: sustained efficacy without rapid attenuation.

### Gene Expression Profiles Across Functional Groups

To examine the transcriptional basis of the observed fitness divergence, we analyzed the expression of 14 target genes across 5 generations (F1 to F5) under Control, DDVP, and EO treatments. Genes were grouped into 4 functional categories (detoxification, antioxidant defense, digestion/metabolism, and lipid/cholesterol metabolism) based on their annotated roles (Figs 3 and 4). Two-way RM-ANOVA confirmed a highly significant Treatment × Generation interaction (*F *= 42.82, *P *< 0.0001), together with significant main effects of Treatment (*F *= 367.5, *P *< 0.0001) and Generation (*F *= 316.1, *P *< 0.0001), indicating that expression trajectories were not parallel across the 3 regimes (Fig. 3).

**Fig. 3.**
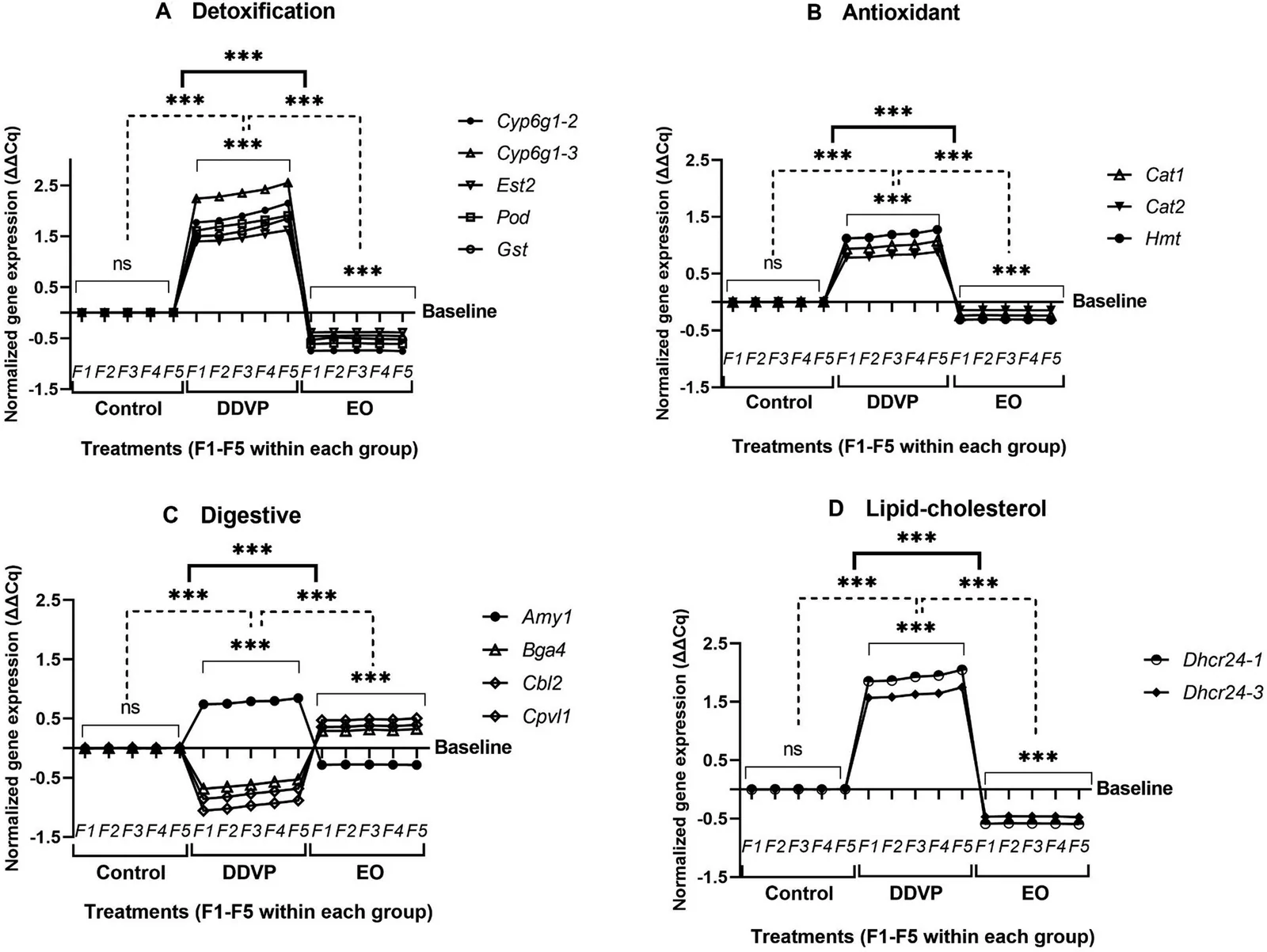
Normalized gene expression (ΔΔCq) of *C. maculatus* across 4 metabolic groups (detoxification (A), antioxidant (B), digestive (C), and lipid–cholesterol (D)) under Control, DDVP, and EO treatments over F1 to F5. Values represent log₂ fold change relative to Control (baseline = 0). DDVP induced pronounced up-regulation of detoxification, antioxidant, and cholesterol-metabolism genes, while EO expression remained near baseline. Digestive genes were initially suppressed by DDVP but recovered by later generations. Significance markers : ***P < 0.001, ns = non-significant.

**Fig. 4.**
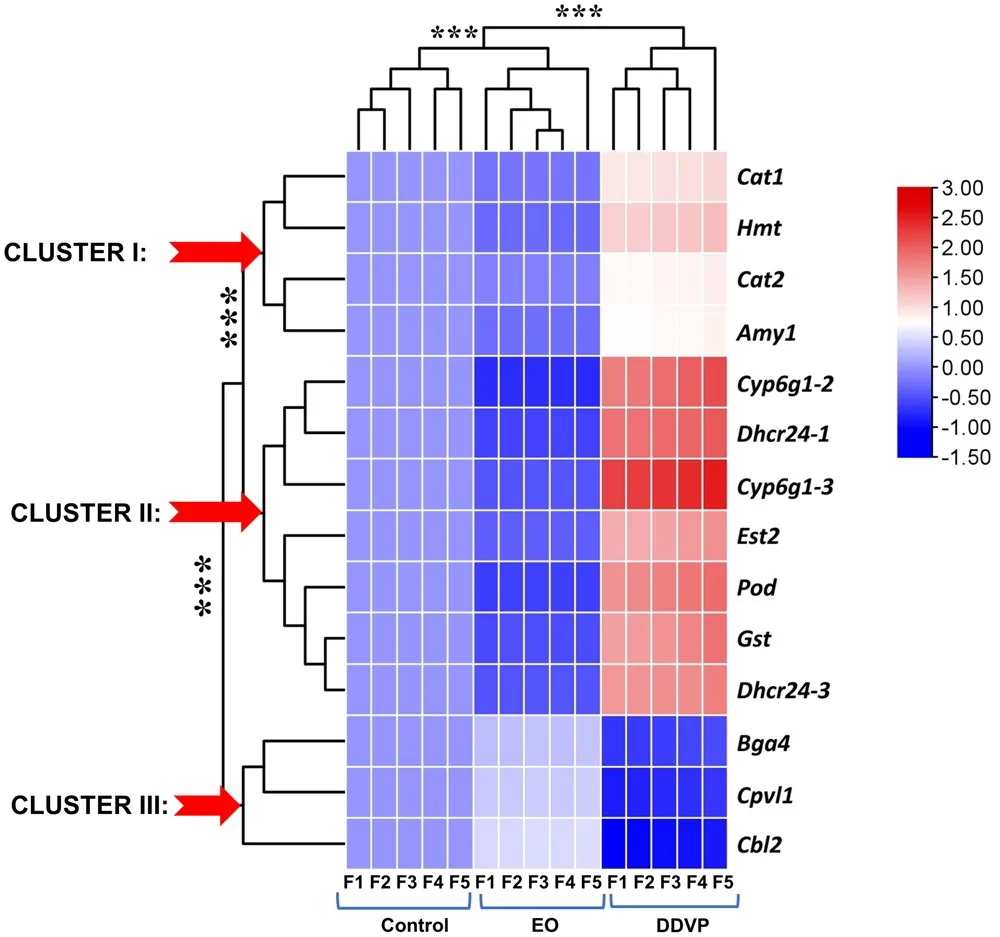
Hierarchical clustering heatmap of ΔΔCq values for 14 candidate genes across 5 generations under Control (Ctrl), essential oil (EO), and DDVP treatments. Blue = downregulation, white = no change, red = upregulation (scale −1.50 to +3.00). Row and column dendrograms used Euclidean distance and complete linkage. Three gene clusters were identified: Cluster I (mild DDVP-responsive: *Cat1*, *Hmt*, *Cat2*, *Amy1*), Cluster II (strongly DDVP-induced: *Cyp6g1-2*, *Cyp6g1-3*, *Est2*, *Pod*, *Gst*, *Dhcr24-1*, *Dhcr24-3*), and Cluster III (DDVP-suppressed: *Bga4*, *Cpvl1*, *Cbl2*). Column clustering separated Control and EO from DDVP generations. Statistical significance was determined by 2-way RM-ANOVA with Tukey HSD (see text for adjusted *P* values). Significance markers: *** *P* < 0.001. .

#### Detoxification Genes

All 5 detoxification genes (*Cyp6g1-2, Cyp6g1-3*, *Est2*, *Pod*, *Gst*) were significantly upregulated under DDVP compared to the Control from F1 onward (all adjusted *P *< 0.0001), with expression increasing progressively across generations (e.g. *Cyp6g1-2*: DDVP_F1 vs. DDVP_F5, adjusted *P *< 0.0001). By F5, mean ΔΔCq values reached approximately +2.0 to +2.5. Under EO treatment, these genes remained at or near Control levels across all generations (all adjusted *P *> 0.05 vs. Control within the same generation), with no evidence of progressive induction (Fig. 3A).

#### Antioxidant Genes

The antioxidant genes (*Cat1*, *Cat2*, *Hmt*) showed moderate but significant upregulation under DDVP compared to the Control (e.g. *Cat1*: Control_F5 vs. DDVP_F5, adjusted *P *= 0.0023; *Cat2*: adjusted *P *= 0.0087; *Hmt*: adjusted *P *= 0.0045). The magnitude of induction was smaller than for detoxification genes (peak ΔΔCq ∼+1.0 to +1.5). Under EO, these genes showed no significant change relative to the Control (all adjusted *P *> 0.05) (Fig. 3B).

#### Digestive Genes

This group showed a divergent pattern. *Amy1* was significantly upregulated under DDVP (F5 vs. Control: adjusted *P *= 0.0342), whereas *Bga4*, *Cbl2*, and *Cpvl1* were significantly downregulated under DDVP compared to the Control (F5: adjusted *P *= 0.0084, *P* = 0.0045, and *P *= 0.0021, respectively). Under EO, *Bga4*, *Cbl2*, and *Cpvl1* showed recovery toward or above Control levels, with *Cpvl1* reaching significant upregulation by F5 (adjusted *P *= 0.0012). This antagonistic pattern was confirmed by a significant Treatment × Generation interaction for all 3 genes (all *P* < 0.0001) (Fig. 3C).

#### Lipid–Cholesterol Metabolism Genes

Both *Dhcr24-1* and *Dhcr24-3* were strongly upregulated under DDVP from F3 onward, reaching peak expression at F5 (vs. Control: both adjusted *P *< 0.0001). Under EO, expression remained near Control levels throughout (all adjusted *P *> 0.05). The Treatment × Generation interaction was highly significant for both genes (both *P *< 0.0001) (Fig. 3D).

#### Cross-Cluster Analysis and Antagonistic Regulation

The hierarchical clustering heatmap (Fig. 4) revealed 3 distinct gene clusters that were statistically validated by Tukey’s multiple-comparisons test (α = 0.05). Cluster I comprised *Cat1*, *Hmt*, *Cat2*, and *Amy1* and showed mild to moderate positive ΔΔCq shifts under DDVP compared with Control and EO, with significant differences observed mainly in later generations (adjusted *P *< 0.05 for DDVP_F4 to F5 vs. Control). Cluster II included *Cyp6g1-2*, *Cyp6g1-3*, *Est2*, *Pod*, *Gst*, *Dhcr24-1*, and *Dhcr24-3* and exhibited the strongest DDVP-associated upregulation, with all 7 genes significantly upregulated under DDVP versus Control from F1 onward (all adjusted *P *< 0.0001). Cluster III contained *Bga4*, *Cpvl1*, and *Cbl2* and showed an opposite pattern, with significantly lower ΔΔCq values under DDVP compared with Control (adjusted *P *< 0.01 to <0.001) and partial recovery under EO. Column clustering separated Control and EO generations, which grouped closely, from DDVP generations, which formed a distinct induced clade. This separation reflects a treatment-specific transcriptional reprogramming dominated by the upregulation of detoxification and cholesterol-metabolism genes under DDVP, a response not elicited by EO.

The antagonistic regulation was quantified using the AI (AI = log₂(FC_*EO*_) − log₂(FC_*DDVP*_). Eight detoxification-related genes showed strong negative AI values (AI < −1.5), confirming that EO and DDVP push expression in opposite directions. Several digestive/metabolic genes (*Bga4*, *Cpvl1*, *Cbl2*) showed positive AI values (AI > 1.0), indicating co-induction by EO relative to DDVP. Detailed AI values for all 14 genes are provided in Table 2.

**Table 2.** Quantitative analysis of antagonistic gene regulation between EO and DDVP treatments in *C. maculatus* across 5 generations

Gene	(FC_DDVP_)	(FC_EO_)	log_2_ (FC_DDVP_)	log2 (FC_EO_)	AI	Regulation class
**Detoxification**						
***Gst***	2.85	0.48	1.511	−1.059	−2.570	Antagonism (Strong)
***Pod***	3.12	0.42	1.642	−1.252	−2.893	
***Cyp6g1-2***	3.85	0.35	1.945	−1.515	−3.459	
***Cyp6g1-3***	2.92	0.52	1.546	−0.943	−2.489	
***Est2***	2.45	0.58	1.293	−0.786	−2.079	
**Antioxidant/stress**						
***Cat1***	1.45	0.72	0.536	−0.474	−1.010	Antagonism (Moderate)
***Cat2***	1.28	0.92	0.356	−0.120	−0.642	Antagonism (Weak)
***Hmt***	2.15	0.65	1.104	−0.621	−1.726	Antagonism (Moderate)
**Digestive**						
***Amy1***	1.65	0.68	0.722	−0.556	−1.279	Co-induction (Moderate)
***Bga4***	0.62	1.48	−0.690	0.566	+1.255	
***Cpvl1***	0.55	1.62	−0.862	0.696	+1.558	Co-induction (Strong)
***Cbl2***	0.48	1.38	−1.059	0.465	+1.524	
**Lipid/cholesterol**						
***Dhcr24-1***	3.56	0.44	1.833	−1.184	−3.017	Antagonism (Strong)
***Dhcr24-3***	2.92	0.52	1.546	−0.943	−2.489	Antagonism (Strong)

FC values calculated via 2^(−ΔΔCq) method (all ≥0). AI = log_2_(FC_EO_)−log_2_ (FC_DDVP_). Negative AI indicates antagonism (EO and DDVP push expression in opposite directions). Positive AI = co-induction (same direction). Classification thresholds: |AI| > 1.5 = Strong, 0.5 to 1.5 = Moderate, <0.5 = Weak.

## Discussion

This study provides a comprehensive molecular profile of *C. maculatus* responses to DDVP and *L. adoensis* EO. The results demonstrate a fundamental dichotomy: DDVP predominantly upregulates detoxification genes, whereas EO treatment is associated with their suppression or basal-level expression.

### DDVP: Upregulation of Detoxification Genes

The upregulation of P450s (*Cyp6g1-2*, *Cyp6g1-3*), GSTs (*Gst*), esterases (*Est2*), peroxidases (*Pod*), and catalases (*Cat1*, *Cat2*) under DDVP aligns with established mechanisms of metabolic resistance, wherein enhanced enzymatic activity facilitates toxin degradation (Hafeez et al. 2022, Zhang et al. 2024). These genes showed consistently positive log₂(FC_DDVP_) values (e.g. *Gst*: 2.954; *Cyp6g1-2*: 1.753; *Cyp6g1-3*: 2.223), confirming a broad inductive response across multiple enzyme classes. From F3 onward, this elevated transcriptional activity was maintained, coinciding with progressive recovery of fitness parameters (survival, fecundity, emergence) in DDVP-treated groups.

Digestive and metabolic genes showed more variable responses under DDVP. *Amy1* was modestly upregulated (log₂(FC_DDVP_) = 0.722), whereas *Bga4*, *Cbl2*, and *Cpvl1* were downregulated (−0.690, −1.059, and −0.862, respectively). These mixed responses likely reflect general metabolic adjustments rather than a dedicated detoxification effort. Similar physiological trade-offs have been observed in other insects under chemical stress (Després et al. 2007, Hafeez et al. 2022), but our data do not support grouping these genes into a single functional unit.

### EO: Association With Transcriptional Suppression of Detoxification Genes

In stark contrast, EO treatment was associated with downregulation or basal-level expression of the same detoxification genes that DDVP induced. Eight detoxification-related genes (*Gst*, *Pod*, *Cyp6g1-2*, *Cyp6g1-3*, *Est2*, *Hmt*, *Dhcr24-1*, *Dhcr24-3*) showed strong antagonistic indices (AI < −1.5), indicating EO pushed their expression in the opposite direction to DDVP. This pattern indicates that EO exposure did not trigger the coordinated detoxification program seen under DDVP, contributing to sustained mortality across generations.

This transcriptional pattern aligns with the fitness data. Across all 5 generations, EO-treated insects showed consistent survival (∼80% to 87%), stable fecundity (∼145 to 152 eggs per female), and flat pupal eclosion (75% to 80%), with no sign of recovery (Figs 1 and 2). Without upregulation of P450s, GSTs, and esterases, EO-exposed weevils remained vulnerable generation after generation. The ability of EO constituents to inhibit transcriptional activation of defense genes has been observed in other systems (Li et al. 2007, Kshatriya and Gershenzon 2024), supporting the notion that certain phytochemicals may interfere with the transcriptional activation of defense genes by modulating upstream regulatory pathways (Amezian et al. 2021). Monoterpenoid-rich EOs have been reported to modulate detoxification gene expression without triggering full resistance (Tak and Isman 2017, Gupta et al. 2023). Our data are consistent with that view.

However, we emphasize that these findings are correlational. We did not test whether EO constituents directly inhibit transcription factors such as CncC/Keap1, nor did we perform functional validation (RNAi, promoter assays, enzymatic activity measurements). Therefore, while the antagonistic expression patterns are consistent with EO-mediated interference with detoxification induction, we cannot conclusively attribute the observed fitness effects to transcriptional suppression alone. Alternative explanations, including differential penetration, pharmacokinetic interactions, or pleiotropic effects, cannot be ruled out.

Interestingly, 3 digestive enzyme genes (*Bga4*, *Cpvl1*, and *Cbl2*) showed positive AI values, indicating co-induction by EO and DDVP. These genes were downregulated by DDVP but upregulated by EO, suggesting the 2 treatments impose different metabolic demands, potentially reflecting a trade-off between detoxification and digestive physiology (Després et al. 2007, Karban 2011).

In summary, under DDVP, detoxification genes were induced and fitness recovered from F3 onward. Under EO, genes remained at basal levels and fitness stayed suppressed. This dichotomy (induction with recovery versus suppression with sustained cost) captures the core finding of this study.

## Conclusion and Future Directions

This study demonstrates that *L. adoensis* EO and DDVP elicit fundamentally different transcriptional and fitness responses in *C. maculatus* across 5 generations. DDVP exposure was associated with the upregulation of detoxification genes and progressive recovery of fitness parameters, consistent with tolerance development. In contrast, EO treatment was associated with suppression of these same detoxification genes and sustained fitness costs across all generations. The antagonistic transcriptional regulation observed between the 2 treatments suggests that EO may interfere with the induction of metabolic resistance pathways.

Several limitations should be acknowledged. Gene expression data represent pooled samples, which may obscure inter-individual variation. The absence of functional validation means the relationship between gene expression changes and fitness outcomes remains correlational. Heritability assays would be required to confirm whether the observed tolerance has a genetic basis. Additionally, the study was conducted under controlled laboratory conditions, and field validation is needed to assess ecological relevance.

Future studies employing RNAi-mediated gene silencing, enzymatic activity assays, and transcriptomic profiling of upstream regulatory pathways would help establish causal links between the observed transcriptional patterns and the sustained efficacy of *L. adoensis* EO. Identifying the specific EO constituents responsible for transcriptional suppression could inform targeted formulation strategies. Despite these limitations, the contrasting multigenerational profiles of DDVP and EO highlight the potential of botanical insecticides for resistance management. The absence of detectable adaptation to EO across 5 generations warrants further investigation into its mode of action and field applicability.

## Supplementary Material

ieag107_Supplementary_Data

## Data Availability

All data supporting the findings of this study are included within this article and its Supplementary Information file. The underlying raw data are available from the corresponding author upon reasonable request.
