# Supplementary material for: Exploring the multigenerational effects of Lippia adoensis (Lamiales: Verbenaceae) essential oil and Dichlorvos on Callosobruchus maculatus (Coleoptera: Bruchidae) life-history traits and gene expression
Source: J Insect Sci. 2026 Sep 29;26(5):ieag107. doi: 10.1093/jisesa/ieag107 (PMC13623516; doi:10.1093/jisesa/ieag107)
Supplement: Supplementary file 1 [file ieag107_supplementary_data.pdf]

Exploring the Multigenerational Effects of *Lippia adoensis* Essential Oil and Dichlorvos on *Callosobruchus maculatus* (Coleoptera: Bruchidae) Life-History Traits and Gene Expression

Electronic Supplementary Materials

I. RNA extraction and cDNA synthesis workflow

A schematic overview of the RNA extraction and cDNA synthesis workflow is provided in the figure below. Total RNA was extracted from dissected gut tissues using TRIzol™ reagent, treated with DNase I to remove genomic DNA contamination, and reverse-transcribed using the PrimeScript™ RT reagent Kit with gDNA Eraser (Takara Bio, Japan).

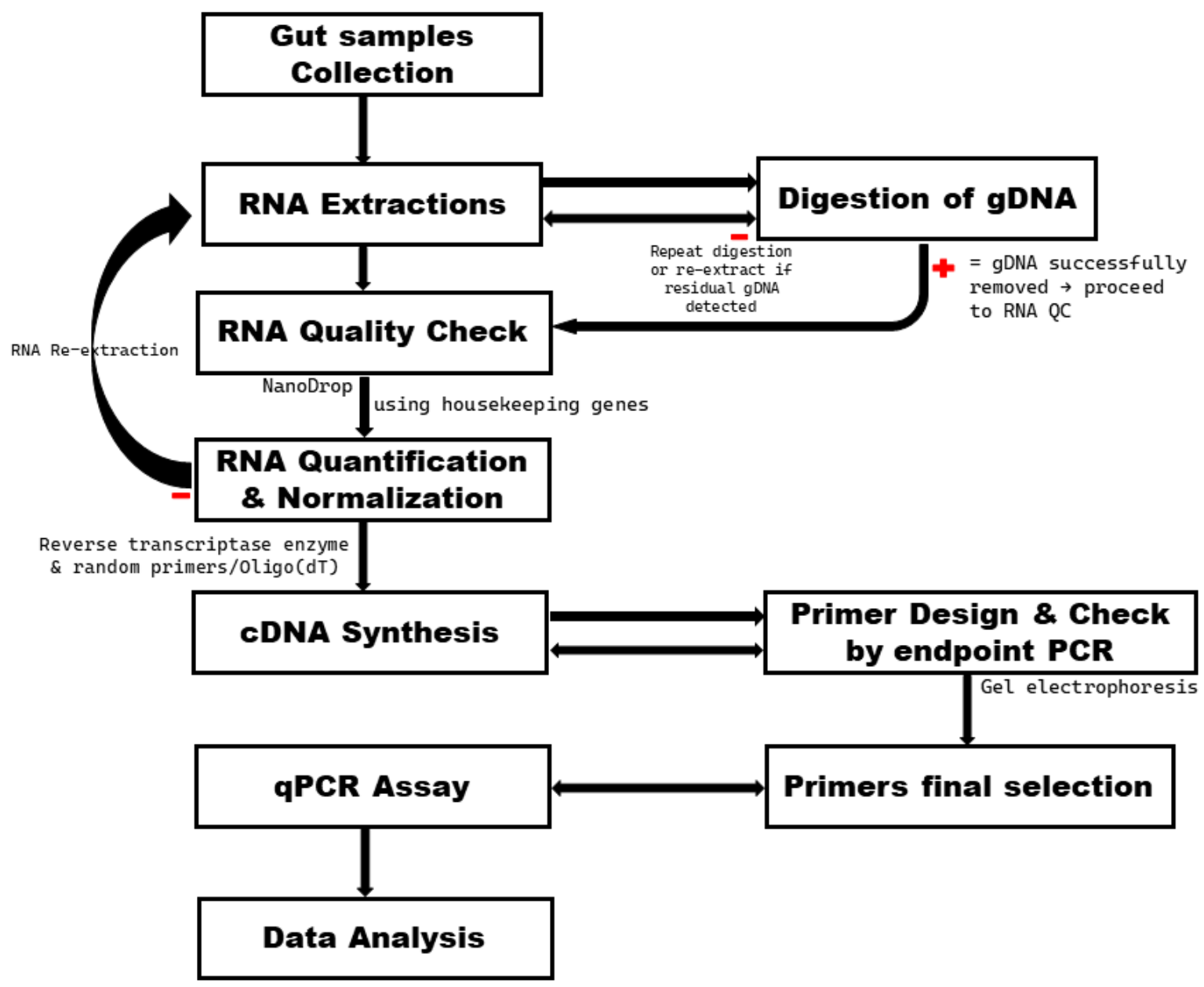

Figure S1: Experimental procedures of qRT-PCR reactions of targeted genes

II. Verification of primer specificity by agarose gel electrophoresis

Representative 1.5% agarose gel image of polymerase chain reaction (PCR) amplicons for the 7 targeted detoxification genes (upper electropherogram) and 7 digestive/metabolic genes (lower electropherogram). The expected amplicon sizes for each gene are indicated. The gel demonstrates a single, sharp band of the correct molecular weight for each primer pair, confirming the specificity of the amplification and the absence of primer-dimers or non-specific products. Lanes: M, 100 bp DNA ladder; subsequent lanes correspond to PCR products for genes *Cat1, Cat2, Cyp6g1-2, Cyp6g1-3, Gst, Est2, Pod, Amy1, Bga4, Cbl2,*

Cpvl1, Hmt, Dhcr24-1, and *Dhcr24-3*. The PCR was performed using cDNA template from *Callosobruchus maculatus*.

Table S1: Primer sequences used for qRT-PCR of targeted genes in *Callosobruchus maculatus*. Ta: Annealing temperature.

Genes names	Sequence source	Primer Direction	Sequence (5'->3')	Amplicon (bp)	Ta (°C)	Efficiency (%)
Rpl32	NM_001011587	Forward	AGTAAATTAAAGAGAAACTGGCGTAA	178	55	98.5
		Reverse	TAAAACTTCCAGTTCCTTGACATTAT	178		
Ef1α	NM_001011628	Forward	GAACATTTCTGTGAAAGAGTTGAGGC	387	55	96.8
		Reverse	TTTAAAGGTGACACTCTTAATGACGC	387		
Cat1	FK669179	Forward	ATCGGCAAAAAGACCCCGAT	82	55	98.5
		Reverse	TGTGTGTGGATGAAGGACGG	82		
Cat2	FK669180	Forward	CAGTCCTATAGCCTGTGGCG	151	55	97.0
		Reverse	CTGGCCCCACTCGGAATATC	151		
Cyp6g1-2	FK669165	Forward	TTCCTGGTCTTGAGCACGTC	130	55	99.5
		Reverse	AAACGTCCCACACGTCGTAA	130		
Cyp6g1-3	FK669166	Forward	ACGTCTGGAAAGCTCAAGGG	184	55	98.8
		Reverse	CATCTTCCCAGGCAGTGAGG	184		
Est2	FK669192	Forward	AGGGGAATGGCGAACAACAT	141	55	100.4
		Reverse	TTGGTGGCATTCCATACCCC	141		
Gst	FK669177	Forward	ACGTTGGCTGACGGAGATTT	114	55	103.7
		Reverse	CTGTTTCCAGCCAGGTAGGG	114		
Pod	FK669176	Forward	TCGGTCTCCCACACGAGTAT	185	55	96.9
		Reverse	GGTGTTGACCGCTACCATGA	184		
Amy1	FK668918	Forward	GCTCCAGGAAGGAACAGCAT	248	55	102.3
		Reverse	ACTCCTGCCTTGTTACACCG	248		
Bga4	FK668907	Forward	TCCTACACGGGGAGTTGCTA	359	55	101.2
		Reverse	GGGTTGTCCCTTGGGGTATG	359		
Cbl2	FK669001	Forward	AGCAAGTCACTAGCACCACC	226	55	97.9
		Reverse	TAGTGCCCCCGTAGACATCA	226		
Cpvl1	FK668971	Forward	CCACCGTTGTTTTTGACCCC	187	55	104.9
		Reverse	GGGCCTTAGCGTAGTCTGTC	187		
Hmt	FK669008	Forward	AGTGGCACCTGCCTAGGATA	174	55	103.2
		Reverse	CGATGCTCTTGCCATCTTGC	174		
Dhcr24-1	FK669017	Forward	TGAGGTAACCGGAAGGGACA	282	55	105.0
		Reverse	CCAACTTGATTTCCGCTGGC	282		
Dhcr24-3	FK669019	Forward	AGTGCTCCGAAACAGCATGA	181	55	99.9
		Reverse	CTGTTTGCCATCCTGGCCTA	181		

<u>Method S1</u>. Cloning and Sequencing for Amplicon Verification

To confirm the identity of the amplicons, PCR products for the gene of interest (*Gst*) were separated on a 1.5% agarose gel. Bands of the expected size were excised, purified using the SanPrep Column DNA Gel Extraction Kit (Sangon Biotech, China), and ligated into the pMD™18-T vector (TaKaRa, China). The recombinant plasmid was transformed into chemically competent *E. coli* DH5α cells. Positive clones were screened by colony PCR, and plasmids from selected clones were Sanger sequenced by TsingKe Biological Technology (Beijing, China) to verify the insert sequence.

<u>Table S2:</u> Quantitative Real-Time PCR (qRT-PCR) reaction conditions and cycling parameters

1- Reaction composition (20 µL total volume):

Component	Volume (µL)
2× TB Green Premix Ex Taq™ II (Tli RNaseH Plus)	10
Forward primer (10 µM)	0.4
Reverse primer (10 µM)	0.4
Diluted cDNA template (1:5)	2
Nuclease-free water	7.2

2- Cycling conditions:

Step	Temperature (°C)	Time	Cycles
Initial denaturation	95	30 s	1
Denaturation	95	5 s	40
Annealing / Extension	55	30 s	40
Melt curve analysis	55 → 95	0.5°C increment / 10 s	1

Instrument: ABI 7300 Real-Time PCR System (Applied Biosystems, USA) TB Green® Premix Ex Taq™ II (Tli RNaseH Plus) (Takara Bio, Japan)

<u>Table S3</u>: Step-by-step calculation of Antagonistic Index (AI) of target genes

Formula: $AI = \log_2(FC_{EO}) - \log_2(FC_{DDVP})$; where **FC =** $2^{(-\Delta\Delta Cq)}$ for each treatment relative to Control.

Gene	(FC_{DDVP})	(FC_{EO})	$Log_2(FC_{DDVP})$	Log_2(FCEO)	AI	Class
Detoxification genes						
Gst	2.85	0.48	ln(2.85)/0.6931= 1.511	ln(0.48)/0.6931= −1.059	−1.059 − 1.511 = **−2.570**	Antagonism (Strong)
Pod	3.12	0.42	ln(3.12)/0.6931= 1.1378/0.6931 = 1.642	ln(0.42)/0.6931= −0.8675/0.6931 = −1.252	−1.252 − 1.642 = **−2.893**	Antagonism (Strong)
Cyp6g1-2	3.85	0.35	ln(3.85)/0.6931= 1.3481/0.6931 = 1.945	ln(0.35)/0.6931= −1.0498/0.6931 = −1.515	−1.515 − 1.945 = **−3.459**	Antagonism (Strong)
Cyp6g1-3	2.92	0.52	ln(2.92)/0.6931= 1.0714/0.6931 = 1.546	ln(0.52)/0.6931= −0.6539/0.6931 = −0.943	−0.943 − 1.546 = **−2.489**	Antagonism (Strong)
Est2	2.45	0.58	ln(2.45)/0.6931= 0.8961/0.6931 = 1.293	ln(0.58)/0.6931= −0.5447/0.6931 = −0.786	−0.786 − 1.293 = **−2.079**	Antagonism (Strong)
Antioxidant / Stress genes						
Cat1	1.45	0.72	ln(1.45)/0.6931= 0.3716/0.6931 = 0.536	ln(0.72)/0.6931= −0.3285/0.6931 = −0.474	−0.474 − 0.536 = **−1.010**	Antagonism (Moderate)
Cat2	1.28	0.82	ln(1.28)/0.6931= 0.2469/0.6931 = 0.356	ln(0.82)/0.6931= −0.1985/0.6931 = −0.286	−0.286 − 0.356 = **−0.642**	Antagonism (Weak)
Hmt	2.15	0.65	ln(2.15)/0.6931= 0.7655/0.6931 = 1.104	ln(0.65)/0.6931= − 0.4308/0.6931 = −0.621	−0.621 − 1.104 = **−1.726**	Antagonism (Moderate)
Digestive / Metabolic genes						
Amy1	1.65	0.68	ln(1.65)/0.6931= 0.5008/0.6931 = 0.722	ln(0.68)/0.6931= − 0.3857/0.6931 = −0.556	−0.556 − 0.722 = **−1.279**	Antagonism (Moderate)
Bga4	0.62	1.48	ln(0.62)/0.6931= − 0.4780/0.6931 = − 0.690	ln(1.48)/0.6931= 0.3920/0.6931 = 0.566	0.566 − (− 0.690) = **+1.255**	Co-induction (Moderate)
Cpvl1	0.55	1.62	ln(0.55)/0.6931= − 0.5978/0.6931 = − 0.862	ln(1.62)/0.6931= 0.4824/0.6931 = 0.696	0.696 − (− 0.862) = **+1.558**	Co-induction (Strong)
Cbl2	0.48	1.38	ln(0.48)/0.6931= − 0.7339/0.6931 = − 1.059	ln(1.38)/0.6931= 0.3221/0.6931 = 0.465	0.465 − (− 1.059) = **+1.524**	Co-induction (Strong)
Lipid / Cholesterol metabolism genes						
Dhcr24-1	3.56	0.44	ln(3.56)/0.6931= 1.2704/0.6931 = 1.833	ln(0.44)/0.6931= − 0.8209/0.6931 = −1.184	−1.184 − 1.833 = **−3.017**	Antagonism (Strong)
Dhcr24-3	2.92	0.52	ln(2.92)/0.6931= 1.0714/0.6931 = 1.546	ln(0.52)/0.6931= − 0.6539/0.6931 = −0.943	−0.943 − 1.546 = **−2.489**	Antagonism (Strong)

Table S4: Complete chemical composition of *Lippia adoensis* essential oil (young leaves; Y_*L. adoensis*) identified by GC–MS (FM3.M INNOWax column). Compounds are listed in decreasing relative abundance.

No.	Compound	Class	RI (exp)	RI (lit)	Retention time (min)	Relative (%)	NIST match (%)	Identification method
1	Thymol	Oxygenated monoterpene	2198	2189	51.98	22.02	97	RI, MS
2	Thymol acetate	Oxygenated monoterpene	1885	1879	41.03	15.21	96	RI, MS
3	*para*-Cymene	Monoterpene hydrocarbon	1275	1280	17.33	13.85	98	RI, MS
4	Triacetin	Hydrocarbon derivative	2045	2038	48.59	9.13	91	RI, MS
5	γ-Terpinene	Monoterpene hydrocarbon	1249	1255	16.13	6.42	99	RI, MS
6	Caryophyllene	Sesquiterpene hydrocarbon	1610	1608	31.29	3.82	97	RI, MS
7	3-Aminopyrazole	Hexane and octane derivative	2598	2590	31.05	3.30	89	RI, MS
8	Carvacrol	Oxygenated monoterpene	2225	2218	39.84	3.26	96	RI, MS
9	Limonene	Monoterpene hydrocarbon	1198	1203	13.47	3.36	98	RI, MS
10	β-Farnesene	Sesquiterpene hydrocarbon	1662	1660	34.67	2.88	95	RI, MS
11	Carvone	Oxygenated monoterpene	2485	2480	36.72	1.88	88	RI, MS
12	α-Terpinene	Monoterpene hydrocarbon	939	932	12.98	1.33	99	RI, MS
13	*m*-tert-Butylphenol	Hexane and octane derivative	1992	1985	35.21	1.19	94	RI, MS
14	α-Thujene	Monoterpene hydrocarbon	930	924	6.63	1.14	97	RI, MS
15	*para*-Thymol	Oxygenated monoterpene	1805	1810	41.80	0.96	95	RI, MS
16	Verbenone	Oxygenated monoterpene	1218	1220	35.95	0.72	99	RI, MS
17	Terpinen-4-ol	Oxygenated monoterpene	1609	1611	31.64	0.63	95	RI, MS
18	α-Pinene	Monoterpene hydrocarbon	979	976	6.43	0.40	98	RI, MS
19	Caryophyllene oxide	Oxygenated sesquiterpene	2178	2172	45.32	0.39	91	RI, MS
20	Linalool	Oxygenated monoterpene	1567	1553	29.47	0.38	98	RI, MS
21	β-Phyllandrene	Sesquiterpene hydrocarbon	1449	1454	14.28	0.36	96	RI, MS
22	Sabinene	Oxygenated monoterpene	975	969	10.36	0.31	97	RI, MS
23	Z-β-Terpineole	Oxygenated monoterpene	1712	1720	25.94	0.19	96	RI, MS
24	E-Limonen oxide	Oxygenated monoterpene	1528	1532	25.64	0.18	97	RI, MS
25	Umbellulone	Oxygenated monoterpene	1528	1532	33.16	0.13	97	RI, MS
26	β-Cubebene	Sesquiterpene hydrocarbon	1395	1398	35.64	0.11	94	RI, MS
27	3-Carene	Monoterpene hydrocarbon	991	988	11.16	0.11	98	RI, MS
28	Fenchone	Oxygenated monoterpene	1835	1842	22.91	0.10	96	RI, MS
29	Z-Ocymene	Monoterpene hydrocarbon	953	950	11.50	0.08	97	RI, MS
30	Terpinolene	Monoterpene hydrocarbon	930	924	17.84	0.08	97	RI, MS
31	Piperitone	Oxygenated monoterpene	1712	1720	43.40	0.08	96	RI, MS
32	α-Caryophyllene	Sesquiterpene hydrocarbon	1389	1392	34.11	0.08	96	RI, MS
33	Eugenol	Oxygenated monoterpene	2245	2240	51.42	0.08	92	RI, MS
34	1-Octen-3-ole	Hexane and octane derivative	1992	1985	25.39	0.07	94	RI, MS
35	Camphor	Oxygenated monoterpene	2095	2090	28.00	0.02	92	RI, MS
36	β-Pipene	Monoterpene hydrocarbon	939	932	9.92	Trace	99	RI, MS
37	α-Phyllandrene	Monoterpene hydrocarbon	1449	1454	12.32	Trace	96	RI, MS
38	2-Methyl-1-butanol	Hexane and octane derivative	2810	2805	—	*Not quantified*	90	RI, MS
39	1-Octen-3-ole	Hexane and octane derivative	1992	1985	—	*Not quantified*	94	RI, MS
40	α-Copane	Sesquiterpene hydrocarbon	1485	1489	—	*Not quantified*	93	RI, MS
41	α-Terpineole	Oxygenated monoterpene	1712	1720	—	*Not quantified*	96	RI, MS
42	Triacetin	Hexane and octane derivative	1992	1985	—	*Not quantified*	94	RI, MS
43	Mytenale	Oxygenated monoterpene	2350	2345	—	*Not determined*	91	RI, MS

Group totals (from source): Hexane and octane derivatives (13.69%); Monoterpene hydrocarbons (26.98%); Oxygenated monoterpenes (46.14%); sum of monoterpenes (Mt): 73.12%; Sth: sesquiterpene hydrocarbons (6.34%); Oxygenated sesquiterpenes (0.39%); sum of sesquiterpenes (St): 6.73%; Trace (< 0.02%); Not quantified; X: not determined; Y_: Young leaves. **Total identified: 93.54%.**
